# Sexual conflict predicts morphology and behavior in two species of penduline tits

**DOI:** 10.1186/1471-2148-10-107

**Published:** 2010-04-23

**Authors:** René E van Dijk, Ákos Pogány, Jan Komdeur, Penn Lloyd, Tamás Székely

**Affiliations:** 1 Department of Biology and Biochemistry, University of Bath, Claverton Down, Bath BA2 7AY, UK; 2 Animal Ecology Group, Centre for Ecological and Evolutionary Studies, University of Groningen, Biological Centre, P.O. Box 14, 9750 AA Haren, the Netherlands; 3 Department of Animal and Plant Sciences, University of Sheffield, Western Bank, Sheffield S10 2TN, UK; 4 Göd Biological Centre, Eötvös Loránd University, Jávorka Sándor u. 14., Göd, H-2131, Hungary; 5 Percy FitzPatrick Institute, DST/NRF Centre of Excellence, University of Cape Town, Rondebosch 7701, Cape Town, South Africa

## Abstract

**Background:**

The evolutionary interests of males and females rarely coincide (sexual conflict), and these conflicting interests influence morphology, behavior and speciation in various organisms. We examined consequences of variation in sexual conflict in two closely-related passerine birds with contrasting breeding systems: the Eurasian penduline tit *Remiz pendulinus *(EPT) exhibiting a highly polygamous breeding system with sexually antagonistic interests over parental care, and the socially monogamous Cape penduline tit *Anthoscopus minutus *(CPT). We derived four *a priori *predictions from sexual conflict theory and tested these using data collected in Central Europe (EPT) and South Africa (CPT). Firstly, we predicted that EPTs exhibit more sexually dimorphic plumage than CPTs due to more intense sexual selection. Secondly, we expected brighter EPT males to provide less care than duller males. Thirdly, since song is a sexually selected trait in many birds, male EPTs were expected to exhibit more complex songs than CPT males. Finally, intense sexual conflict in EPT was expected to lead to low nest attendance as an indication of sexually antagonistic interests, whereas we expected more cooperation between parents in CPT consistent with their socially monogamous breeding system.

**Results:**

Consistent with our predictions EPTs exhibited greater sexual dimorphism in plumage and more complex song than CPTs, and brighter EPT males provided less care than duller ones. EPT parents attended the nest less frequently and less simultaneously than CPT parents.

**Conclusions:**

These results are consistent with sexual conflict theory: species in which sexual conflict is more manifested (EPT) exhibited a stronger sexual dimorphism and more elaborated sexually selected traits than species with less intense sexual conflict (CPT). Our results are also consistent with the notion that EPTs attempt to force their partner to work harder as expected under sexual conflict: each member of the breeding pair attempts to shift the costs of care to the other parent. More brightly colored males benefit more from desertion than dull ones, because they are more likely to remate with a new female. Taken together, the comparison between two closely related species with contrasting breeding systems suggest that sexual conflict over care has influenced the evolution of behavior and morphology in penduline tits.

## Background

Reproduction has long been viewed as a cooperative exercise between male and female partners. Yet, the evolutionary interests of males and females are often different (sexual conflict, [1]). Only in the rare case of semelparity, or when there is full and lifelong monogamy of the pair members will the interests, e.g. in mating rate or amount of parental care, be equal for both sexes [2,3]. Only recently, however, have researchers started to explore the implications of sexual conflict on speciation, breeding systems, and evolution of various life-history traits [4-7]. Sexual conflict is a potent evolutionary force that may mold morphology and behavior [8,9], and promote speciation [10]. For instance, behavioral traits of dung fly, *Sepsis cynipsea*, in populations undergoing intense sexual conflict diverged to a greater extent than flies under relaxed conflict, resulting in different levels of reproductive isolation [11]. Extra-pair copulations in monogamous passerines may also result from sexual conflict. The negative selection for direct benefits from extra-pair copulations for females appears to be greater than the positive selection for indirect benefits, which supports the notion that extra-pair copulations reflect pre-zygotic sexual conflict [12].

Conflicts between parents over care (post-zygotic sexual conflict, [13]) emerge via a trade-off between parental effort and alternative mating opportunities for each parent. As a result, each parent is expected to avoid the costs of care and shift those costs to its partner [5,14]. This may happen through a continuous adjustment of parental effort in response to the mate's current effort (best response rule, [15]), or through a discrete decision to either care for the offspring or to desert the partner and offspring [16,17]. Conflict over care typically occurs when there is an opportunity to reduce parental contribution. For example, a parent may desert the brood when one parent is sufficient to successfully raise the offspring [17,18]. This may occur when resources are plentiful [19] or when offspring require little care, as is often the case with precocial young [20,21]. By deserting, the parent may benefit from finding a new mate and breeding again, thereby enhancing its reproductive success ([3,22,23], but see: [24]). With increasing levels of polygamy, variance in reproductive success increases and thus polygamous breeding systems are usually associated with intense sexual selection [24-28]. Subsequently, sexual selection is expected to act stronger in species experiencing greater disparity in care provisioning.

Here we test *a priori *predictions of sexual conflict theory about the impact of sexual conflict on morphology and behavior by comparing two closely related species of penduline tits [29,30]: the sequentially polygynandrous Eurasian penduline tit *Remiz pendulinus *(henceforth EPT) and the socially monogamous Cape penduline tit *Anthoscopus minutus *(henceforth CPT).

**Figure 1:**
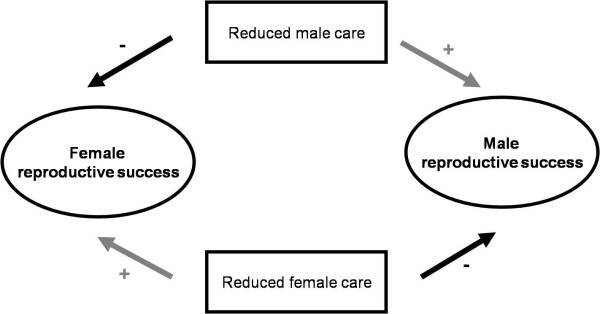
**Sexual conflict in Eurasian penduline tits (after **[23]**)**.

The EPT is a small passerine (body mass about 9 g) with a widespread distribution across Europe and Central Asia. Intense conflict between parents is indicated by several studies that showed that parental care is carried out by a single parent only (either the male or the female) [23,31,32]. In addition, about one third of clutches is deserted naturally by both parents; a pattern consistent between five European populations [23,31,33-35]. Both polygyny and polyandry are common, and since the deserted parents often obtain new mates both sexes may mate with up to six partners in a single breeding season. By deserting the clutch both males and females enhance their own reproductive success, whereas caring reduces reproductive success in both sexes ([23,36]; Figure 1). In contrast, the CPT (body mass about 6 g, endemic to southern Africa) is socially monogamous, and parents cooperate to incubate the eggs and rear the brood together, sometimes assisted by helpers at the nest [36-38]. The pair usually stays together throughout and sometimes across breeding seasons (Lloyd P, van Dijk RE, Pogány Á unpublished data).

Firstly, given that EPT is frequently polygamous and thus likely experiences a larger variance in reproductive success, we predicted more intense sexual selection in EPT than in the socially monogamous CPT. This is expected to result in a stronger sexual plumage dimorphism and more complex song in EPT than in CPT. Both song and plumage are often sexually selected traits in birds, including penduline tits ([39-41], Pogány, Á. et al. unpublished data).

Secondly, we tested the prediction that male EPTs with a wider eye-stripe, which signals male attractiveness [42,43], desert the nest more often than males with a narrower eye-stripe, thus imposing the costs of parental care on their mate. We expected that males with wider eye-stripes desert more frequently than males with narrower ones, since males with wider eye-stripes more often and more quickly procure new mates after desertion [40]. As such, males with wider eye-stripes should derive greater benefits from desertion to offset the potential cost of biparental desertion. Females mated to males with wider eye-stripes, however, face the costs of care and/or reduced reproductive success ([23]; Figure 1).

Finally, nests of both species are sophisticated structures (see below) and built by both sexes. Following predictions from sexual conflict theory (e.g. [5]), however, we expected that in EPT, in which nest desertion is common, parents will attempt shifting the costs of care to their mate. Nest desertion by both males and females takes place at around the third day of the egg laying phase [44-46] and the process of desertion appears to be rapid in our population during which either the male or the female may desert first [36,45]. This is consistent with the prediction from sexual conflict theory that parents attempt to force their mate to work harder [47]. Given the intense conflict in EPT [23], a parent may abstain from building a nest expecting its mate to make up the shortfall. Specifically, we predicted EPT pairs to attend the nest less frequently and less synchronously during the egg-laying phase than in CPT, in which parents are expected to cooperate over nest attendance. In the cooperating CPT we predicted synchronous nest attendance and coordinated nest building by both parents.

## Results

### Plumage

**Figure 2:**
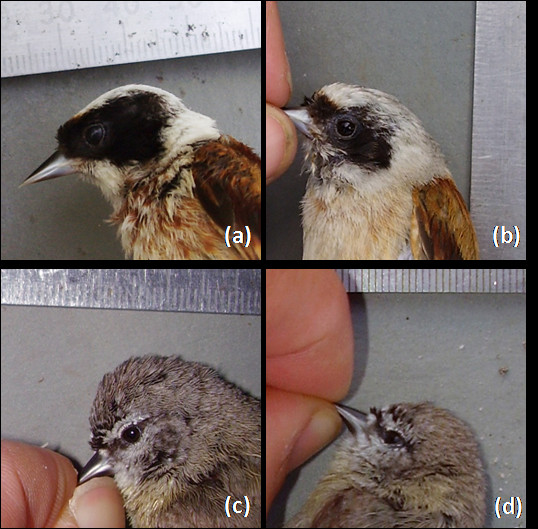
**The eye-stripes of (a) male and (b) female Eurasian penduline tits, and (c) male and (d) female Cape penduline tits**. (see also Table 1).

Consistently with the prediction, in EPT, the eye-stripes of males were significantly (28%) larger than that of females, whereas in CPT the size of the eye-stripe was not different between males and females (Figure 2; sex: *F *= 9.881, *P *= 0.002; species: *F *= 295.358, *P *< 0.001; interaction sex × species: *F *= 10.290, *P *= 0.002, *N *= 206 individuals; Table 1).

**Table 1:** Eyestripe-size of male and female penduline tits.

	Males (cm^2^)	Females (cm^2^)		*P*	*d*	1-β	*N*_required_	*N*_*d*,1-β_
EPT	1.29 ± 0.23 (*N *= 155)	0.93 ± 0.20 (*N *= 34)	*t *= 8.419	<0.001	1.594	> 0.99		
CPT	0.13 ± 0.02 (*N *= 9)	0.14 ± 0.03 (*N *= 8)	*Z *= 0.627	0.531	0.034	0.05	13581	8

*d *= Cohen's effect size, 1-β = power. The sample size required for a statistically significant difference is provided for CPT given the effect size *d *of the underlying data of CPT and the power 1-β set at 0.8 (*N*_required_), and given the effect size *d *in EPT and the power 1-β set at 0.80 (*N*_*d*,1-β_) (see [70]).

**Figure 3:**
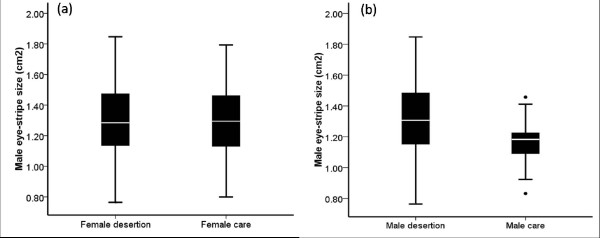
**(a) The eye-stripes of males for whose offspring the female cared (*N *= 48) are not larger than of those males that were deserted by the female (*N *= 73), whereas (b) Eye-stripes of deserting male Eurasian penduline tits (*N *= 104) are larger than the caring ones (*N *= 17)**. Boxplots show median, interquartile range, outliers and extreme cases.

Male EPTs with large eye-stripes deserted their first clutch more often than those with small and thus less attractive eye-stripes (Figure 3a; binary logistic regression model; model effect estimate ± SE = 2.647 ± 1.226, Wald = 4.661, *df *= 1, *P *= 0.031, *N *= 121 males). Females, however, did not care more often for clutches of males with large eye-stripes (Figure 3b; 0.222 ± 0.786, Wald = 0.080, *df *= 1, *P *= 0.778, *N *= 121 males).

### Song

From the song recordings of EPT (127.5 ± 48.4 min (mean ± SD), 16 males) 2229 syllables were analyzed. Adding all recordings from all males together, the total number of different syllables sung in the population ('repertoire size') in EPT did not increase after 46% of the total recording time. Additionally, after the first 52% of recorded syllables (i.e. the first hour of recording from all 16 males) we obtained 14 out of the 16 different syllables we recorded in total (i.e. 88%). All different syllables sung by an individual male were obtained after 71% ± 24% of the total number of syllables recorded per individual. We may have underestimated the repertoire size for individual males, although this would only make our results more conservative (see below). The song recorded from CPT (220.9 min ± 94.3 min, 9 males) contained a total 1918 syllables. We did not find variation in the number of different syllables sung by CPT, so we are confident that we obtained the full repertoire size for CPT.

**Figure 4:**
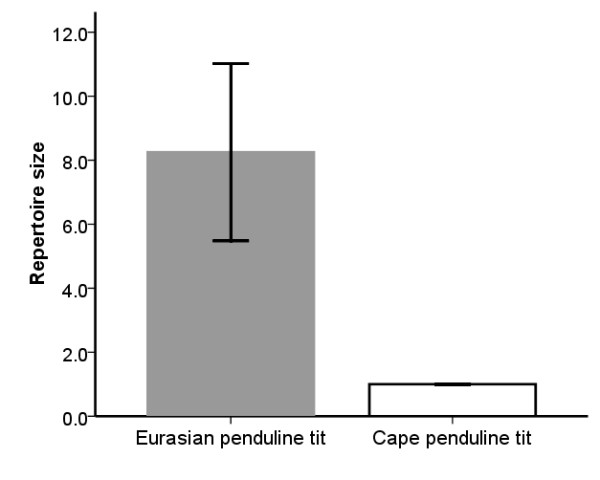
**Repertoire size, i.e. the mean number of different syllables sung by each male, in Eurasian (*N *= 16 males) and Cape penduline tit (*N *= 9 males)**. Bars represent mean ± SD.

The song output was not significantly different between species: EPT males sang 62.8 ± 32.0 (*N *= 16 males) syllables per hour at the nest, whereas CPT males sang 54.0 ± 42.8 (*N *= 9 males) syllables per hour (*t *= 0.581, *P *= 0.567, *N *= 25 males, *d *= 1.461, 1-β = 0.92). EPTs used 8.3 ± 2.8 different syllables (*N *= 16 males), whereas song was invariably mono-syllabic in CPT (*N *= 9 males; Figure 4; one-sample *t*-test with test value = 1; *t *= 10.474, *P *< 0.001, *d *= 3.029, 1-β > 0.99).

### Nest attendance

**Figure 5:**
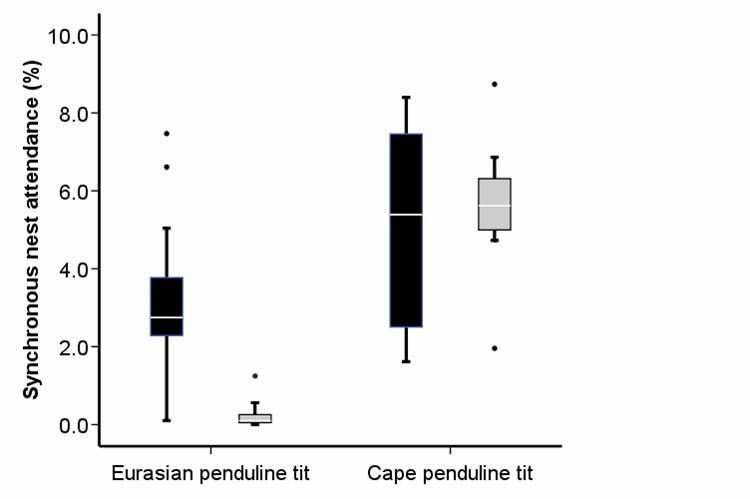
**Expected (black boxes) and observed (shaded boxes) synchronous nest attendance by Eurasian (*N *= 21) and Cape penduline tit (*N *= 7) pairs**. Boxplots show median, interquartile range, outliers and extreme cases.

Eurasian and Cape penduline tits differed significantly in the frequency of synchronous nest attendance by male and female (0.20 ± 0.28% (*N *= 21 pairs) versus 5.56 ± 2.07% (*N *= 7 pairs), respectively; Figure 5; Mann Whitney U; *Z *= ± 3.902, *P *< 0.001, *N *= 28 pairs, *d *= 2.949, 1-β >0.99). This result was corroborated by comparing the absolute time spent at the nest by male and female jointly (EPT: 115s ± 162s, CPT: 2343s ± 864s; Mann Whitney U; *P *< 0.001).

Comparing the expected *versus *observed times at the nest by both parents, we found a significant effect of species (Figure 5; *F *= 20.366, *P *< 0.001, *N *= 28, *η*^2 ^= 0.439, 1-β = 0.99): EPTs spent significantly less time together at the nest than expected by chance (Figure 5; 0.20% *versus *3.23% of time, respectively;*Z *= ± 4.015, *P *< 0.001, *N *= 21 pairs, *d *= 2.898, 1-β > 0.99), whereas in CPT the expected *versus *observed times were not different (Figure 5; 5.56% *versus *5.05% of time, respectively; *Z *= ± 0.845, *P *= 0.398, *N *= 7 pairs, *d *= 0.329, 1-β = 0.09, *N*_required _= 146, *N*_*d*,1-β_; = 4). Finally, EPTs spent significantly less time at the nest (36.0 ± 9.9%, *N *= 21 pairs) than CPTs (49.4 ± 15.7%, *N *= 7 pairs; *F *= 7.075, *P *= 0.013, *η*^2 ^= 0.214, 1-β = 0.726).

## Discussion

Penduline tits (Remizinae) are emerging as one of the model systems in investigations of parental conflict [4,23,31,48]. Here we found support for several *a priori *predictions of sexual conflict theory by comparing the behavior and plumage dimorphism of two closely related species. Firstly, we found substantial sexual plumage dimorphism in EPT, but not in CPT. Secondly, we found that EPT males had a more complex song (i.e. a larger song repertoire) than CPT males. These results together suggest that sexual conflict influences the evolution of plumage dimorphism and complexity of song through intensified sexual selection. We realize that the power to detect a significant difference between the sexes of CPT in size of the eye-stripe is low. However, the sample size required to detect a sexual dimorphism in the size of the eye-stripe in CPT, given the effect size and power (*N*_required_), is unrealistically large (13581), yet with our sample we would have been able to detect a sex difference in eye-stripe size in CPT if it had been of a similar intensity as in EPT (*N*_*d*,1-β_; Table 1). Therefore eye-stripes are sexually monomorphic in CPT. We note that EPT males and females can easily be distinguished in the field, but not CPTs (see various field guides), and we thus suspect our results would be robust even with larger samples sizes.

Thirdly, we found that EPT males with wider eye-striped were more likely to desert their mate than males with narrower eye-stripes. However, females mated to males with wider eye-stripes did not provide care more often than those females that were mated to males with narrower eye-stripes. This somehow contradicts the prediction of the differential allocation hypothesis, which predicts that females mated with more attractive males are willing to invest more in the offspring than females mated to less attractive males [49,50]. One interpretation is retaliation by the females to avoid being exploited by the males: if an attractive male deserts, a female may still desert, despite the loss of the eggs (see also [3,32]). Nevertheless, although females may obtain direct and/or indirect benefits from mating with attractive males, they also pay the full costs of caring or, in case they desert too, their efforts invested in nest building and egg-laying appear to be in vain. This points to the dilemma of EPT females: by choosing an attractive mate she may actually lose [8].

Sexual conflict may be associated to a process of manipulation by one parent and resistance by the other. This potentially affects the evolution of various traits [2,8], and may also explain the difference in sexual dimorphism and song complexity between the two species of penduline tit. Evidence for this arms race between male and female partners derives from pre-copulatory sexual conflict where males are harmful to females during copulation [2,9,51,52]. Males may, for instance, cause genital damage to the female [51], or force the female to mate at a suboptimal rate [4,6,9,53,54]. Males may also try to exploit the female's perception system during mate choice and parental investment, for instance through exaggerated sexually selected traits in males, such as ornaments or song. Females are expected to counter-adapt through more selective mate choice [4,6,8] leading to female resistance to mating and the evolution of exaggerated male display to overcome this resistance ('sexually antagonistic coevolution', [8,55]; see also [56,57]). In that light, the evolutionary driving force of preference is resistance to male-imposed costs, rather than gaining benefits from mating with preferred males, as described under classic sexual selection [6,8,55]. Mediated by such a dynamic process male EPTs may try to manipulate their partner via elaborate plumage and song. This, in conjunction with resistance by the female, may have led to the exaggeration of those traits in EPT, but not in CPT.

An alternative explanation for the elaboration of traits in EPT is the higher population density than in CPT and thus more intense selection driven by male-male competition [58,59]. However, this argument has been challenged [60,61] and breeding density itself is not a selective process, but rather an environmental trait that amplifies or de-amplifies sexual conflict. For instance, more potential mates may be available when breeding densities are high and a parent may then benefit more from desertion rather than caring for the offspring. We believe sexual conflict is a more parsimonious explanation than the alternatives, because our previous work showed strong evidence of intense sexual conflict in EPT [23] and the intensity of sexual conflict is unrelated to breeding density in EPT [35].

Finally, we found that EPT parents not only spend less time overall on nest attendance than CPT, but they also were at the nest less synchronously. The latter was also true when we compared the estimated absolute time the parents spent jointly at the nest, confirming that the difference in day length between our two study sites does not alter our results. These results suggest that EPT parents appear to avoid each other at the nest - consistent with our argument above that one parent may force the partner to work harder. An alternative explanation for the observed pattern in EPT is role division so that EPT partners may take over the job of nest building from each other rather than actively avoiding each other at the nest. This would also result in less time spent together at the nest, albeit that this should be interpreted as a more cooperative behavior as opposed to avoidance due to conflict. The fact that the total time spent on nest attendance (total frequency of individual and joint attendance) is lower in EPT than in CPT, however, corroborates the idea that they actively try to avoid each other, rather than taking over each other's work as a cooperative effort (see [13]).

An alternative hypothesis, mate guarding [62-64], predicts the opposite pattern to what we found: due to frequent mate change and dense breeding population in EPT, one would predict more intense mate guarding in EPT than in CPT and thus more synchronous appearance at the nest.

We acknowledge alternative selective processes to sexual conflict that may influence the evolution of morphology and behavior by acting themselves or acting with sexual conflict. To establish the generality of these results and to test alternative hypotheses, we need phylogenetic comparative studies using the appropriate framework. We are currently working on the first comprehensive phylogenetic hypotheses for Remizinae (van Dijk et al, in preparation), which will serve as backbone for future analyses [65-67].

## Conclusions

We tested *a priori *predictions from sexual conflict theory using two closely-related species of penduline tits that exhibit different breeding systems. Differences in behavior and morphology between the two species were consistent with the predictions of sexual conflict theory. Although various studies have found support for these predictions in relation to pre-zygotic sexual conflict (sexual conflict over mating), whether the same is true for sexual conflict exhibited after fertilization was hitherto unclear. Detailed data from the field, collected from multiple, closely-related species exhibiting a variety of breeding systems in various habitats will further advance this field. The diverse breeding systems of penduline tits are an excellent model system to understand how sexual conflict and cooperation may have shaped the evolution of morphology, behavior, neuro-endocrinology and genome of organisms.

## Methods

### Study sites and data collection

We studied EPTs between April and August in five consecutive breeding seasons (2003-2007) in a reed marsh at a 1321ha fishpond system, Fehértó, in southern Hungary (46°19'N 20°6'E), where approximately 60-90 males and 45-50 females bred each year. We studied eight and six breeding pairs of CPT in September 2006 and 2007, respectively, in coastal scrubland at the 572ha Koeberg Nature Reserve near Cape Town, South Africa (33°40'S 18°26'E). The low number of monitored nests in CPT compared to EPT is due to the lower population density in CPT, as large territories are used by family groups [37]. Both species build similar, domed nests, initiated by the male. In EPT males are unpaired, whereas most CPT males are paired at the onset of building. The nest is finished and maintained jointly by both male and female after pair formation in both species. The egg-laying phase is initiated at a similar stage of nest building, i.e. when the parents start building the entrance tube to the nest.

We searched both study areas for nest-building penduline tits, and visited each nest about every other day to determine which parent attended the nest [32]. At each EPT nest we recorded the date of pair formation. We considered a male to be mated as soon as the pair was seen copulating near the nest or when male and female were seen to build the nest together. For time in season we used a date format as the number of days since 1 April in each year. We trapped and banded birds with one numbered metal band from the Hungarian Ornithological Institute (EPT) or the South African Bird Ringing Scheme (CPT), and a unique combination of three color bands (A.C. Hughes, Middlesex, UK). Three digital photographs were taken of each side of the bird's head using an Olympus FE-100 and a Fujifilm FinePix A203 digital camera. In all photographs we kept a ruler in the background as a reference to measure the size of the eye-stripes. The birds were hand held touching the ground and the camera was positioned at an approximately fixed distance (about 20 cm) from the bird to standardize aberrations. The area of the eye-stripe (to the nearest 0.01 cm^2^), signaling attractiveness in EPT [42,43], was quantified from the digital photographs using Adobe Photoshop 7.0. We took the average of the three measurements for the size of the eye-stripe.

The song of 16 male EPTs was recorded in 2006 for 127.5 ± 48.4 min (mean ± SD) at a randomly selected time of day between 06:28 and 17:50 (CET), using a Marantz PMD 660 portable digital recorder with a Sennheiser ME66 directional microphone. It is worth noting here that Eurasian penduline tits, as well as other species of penduline tits in the genus *Remiz*, sing little compared to other species of song birds and do not engage in the dawn or dusk chorus (van Dijk, RE, Bot, S, and Pogány, Á, pers. obs.). Using the same equipment as for EPT, we recorded the song of 9 CPT males (recording time 220.9 min ± 94.3 min). All recordings for CPT were made during the morning (06:20 - 11:30 UTC). For both species, in the analyses we only included song recordings from mated males. Sonograms of the recordings were created and analyzed using Audacity v. 1.2.6 and Avisoft-SASLab Light v. 3.74.

To investigate nest attendance during nest building, which continues through the laying period, we filmed nests in 2006 and 2007 in both EPT and CPT using a time-lapse video camera (Sony digital handycam, DCR-HC44E) storing one frame every five seconds. In CPT we knew precisely the date when the first egg was laid, and nest attendance of parents was recorded during the second and third days of egg-laying (547 min ± 82 min per day, *N *= 7 pairs). In EPT egg-laying dates were often not known, therefore we recorded nest attendance from after pair formation and during egg-laying for EPT (329 min ± 184 min per day, *N *= 21 pairs), i.e. a more extended period than for CPT. The period before egg-laying involves more nest building than maintenance. We anticipate that this would not influence our results, since the parents are expected to spend more time at the nest during nest building than during nest maintenance, which would result in more nest attendance in EPT than in CPT. The pattern we predicted and found is opposite to this (see Results). Recordings were analyzed frame by frame using MATLAB v. 6.5 (256240 and 96632 frames in total for EPT and CPT, respectively), coding nest attendance (i.e. presence of bird on or inside the nest) as: (i) male-only, (ii) female-only, (iii) joint nest attendance by male and female, or (iv) both parents absent. When EPT parents are inside the nest, the head is still visible. CPT parents often close the entrance spout when they have entered the nest and when they leave it again. This was visible on our video recordings. We were thus able to accurately score the time the parents spent on as well as inside the nest. Behavioral observations carried out for a previous study showed that EPT parents are rarely near the nest together (for instance, one parent building at the nest with the partner perching nearby), so this will not have confounded our results (van Dijk, RE, Szentirmai, I, Székely, T unpublished data). CPT parents, however, are often together near the nest, as is shown by our results.

To distinguish male and female parents from intruders we used individual differences in plumage [43,68], behavior (e.g. females are more often and for longer periods inside the nest than males; intruders are often on the outside of the nest and build very little), and color bands. Ambivalent records, i.e. when the identity of an individual was ambiguous, were excluded (7.1% and 4.8% of total records of EPT and CPT, respectively). These excluded frames consisted of those frames where an individual was present plus all following frames with none of the parents present until a frame where a new bird appeared that could be identified. The aim of latter was to avoid a bias towards estimated absence. As nest desertion takes place during egg-laying in EPT, we only included pre-desertion records.

### Data analyses

We used binary logistic regression models with backward elimination to predict parental care strategy (male or female as response variable; care/desert) at the first clutch of EPT in response to the size of the male eye-stripes. The initial model included year as a categorical covariate and mating date as a continuous covariate. Neither covariate contributed significantly to the model (*P *> 0.255), so both were removed from the final model. The final model provided an adequate fit to both male strategy (Hosmer-Lemeshow goodness-of-fit; χ^2 ^= 10.289, df = 8, *P *= 0.245), and female strategy (χ^2 ^= 6.400, df = 8, *P *= 0.603).

All CPTs videotaped were color banded. The analyses for nest attendance by CPTs included one male that was recorded at two nests in consecutive years with a different female, so we included these as two data. Out of the 21 EPT nests filmed, one male and 18 females were not color banded. Adult returning rates between years are low (5% for males, 2% for females; [69]), therefore it is unlikely that we observed the same unbanded individuals in different years. Additionally, of eight unbanded females in 2006 and the ten in 2007, three and six bred simultaneously, respectively, and we can thus be certain that these are different individuals. For the remaining nine females we cannot exclude the possibility of pseudoreplication, although we suspect it is unlikely given (i) the size of our breeding population and (ii) that the composition of pairs was nearly always different (out of 194 pairs that produced a clutch, only six pairs remained together and produced a second clutch between 2002 and 2007). Pseudoreplication in the plumage analyses was avoided by randomly choosing one measurement per individual.

To examine the degree of synchrony in nest attendance by male and female, i.e. male and female being together at the nest simultaneously, we first calculated the time that the male and female can be expected to spend together at the nest by chance, by multiplying the total percentage nest attendance by the male, i.e. male-only attendance plus attendance by male and female together, with the total percentage nest attendance by the female. We then compared the difference between observed and expected patterns of nest attendance by both species using a General Linear Model (GLM). A GLM was also used to compare the total proportion of time the parents spent at the nest, i.e. the sum of male-only, female-only and joint nest attendance, between the two species. Both GLMs included year as a factor and the first day of filming as a covariate, although neither contributed significantly to either of the models (*P *> 0.138) so they were excluded from the final models. Day lengths are different between Hungary (15 h 46 min ± 0 h 11 min, Budapest) and South Africa (11 h 41 min ± 0 h 12 min, Cape Town) and to test whether this might confound our results we estimated the absolute time the parents attended the nest per day as the percentage of time spent at the nest × day length (day lengths for both study sites collected from http://www.timeanddate.com). We then compared whether the absolute time spent at the nest by both parents is different between EPT and CPT.

We provide effect sizes [70] and power analyses, and applied the asymptotic relative efficiency when estimating power of Mann-Whitney U-tests [71]. If the power of the statistics was relatively low for CPT (i.e. 1-β ≤0.5), we provide the sample size that would be required to find a statistical significant difference between the two groups given Cohen's effect size *d *of the underlying data of CPT and power 1-β = 0.8 (*N*_required_), and the required sample size given the effect size *d *in EPT and power 1-β = 0.8 (*N*_*d*,1-β_). All statistical analyses were performed using SPSS 14.0.0 (SPSS Inc., USA), except power analyses, which were carried out in R (R Development Core Team 2005). We provide mean ± SD, and two-tailed probabilities.

## Authors' contributions

REvD contributed to the design of the study, to the acquisition of data, carried out the statistical analyses and drafted the manuscript in partial fulfillment of a doctoral degree at the University of Bath (United Kingdom). AP contributed to the design of the study and acquisition of data and has been involved in the revision of the manuscript draft in partial fulfillment of a doctoral degree at the Eötvös University (Hungary). JK has commented on earlier versions of the manuscript and has helped towards the acquisition of data. PL has collected data and commented on earlier drafts of the manuscript. TS designed the study and has edited and revised earlier versions of the manuscript. All authors have read and approved the final manuscript.
